# Genome Sequences of 23 SARS-CoV-2 Omicron-Lineage Strains from Bangladesh

**DOI:** 10.1128/mra.00950-22

**Published:** 2022-12-06

**Authors:** Mohabbat Hossain, Omar Hamza Bin Manjur, Lincon Hasda, Mohammad Tanbir Habib, Mokibul Hassan Afrad, Manjur Hossain Khan, Nandita Banik, Nawroz Afreen, Ahmed Nawsher Alam, Mustak Ibn Ayub, Mohammed Ziaur Rahman, Mustafizur Rahman, Farhana Khanam, Sayera Banu, Nicholas R. Thomson, Tahmina Shirin, Firdausi Qadri

**Affiliations:** a Institute for Developing Science and Health Initiatives, Dhaka, Bangladesh; b International Centre for Diarrhoeal Disease Research, Dhaka, Bangladesh; c Institute of Epidemiology, Disease Control, and Research, Dhaka, Bangladesh; d Department of Genetic Engineering and Biotechnology, University of Dhaka, Dhaka, Bangladesh; e Wellcome Sanger Institute, Wellcome Genome Campus, Hinxton, Cambridgeshire, United Kingdom; f London School of Hygiene and Tropical Medicine, London, United Kingdom; DOE Joint Genome Institute

## Abstract

We announce the coding-complete genome sequences of 23 severe acute respiratory syndrome coronavirus 2 (SARS-CoV-2) Omicron strains obtained from Bangladeshi individuals. The Oxford Nanopore Technologies sequencing platform was utilized to generate the genomic data, deploying ARTIC Network-based amplicon sequencing.

## ANNOUNCEMENT

A novel severe acute respiratory syndrome coronavirus 2 (SARS-CoV-2) (family *Coronaviridae*, genus *Betacoronavirus*) variant, known as the Omicron variant (B.1.1.529), was initially reported to the World Health Organization (WHO) on 24 November 2021 (1). Now this variant of concern (VOC) includes more than 100 sublineages (2). Omicron BA.2 sublineage BA.2.75 was first identified in India, and findings showed that BA.2.75 replicates more efficiently in the lungs than does BA.2 or BA.5 (3).

As part of a nationwide coronavirus disease 2019 (COVID-19) surveillance program (Institute of Epidemiology, Disease Control, and Research [IEDCR] protocol IEDCR/IRB/2020/11), nasopharyngeal swab specimens were obtained from routine diagnostic samples from patients across Bangladesh. Reverse transcription (Real-time)-PCR was performed using a commercially available novel coronavirus (2019-nCoV) nucleic acid diagnostic kit (Sansure Biotech, China). A total of 48 SARS-CoV-2 RT-PCR-positive samples were subjected to sequencing. Viral RNA was extracted from nasopharyngeal swab samples using the QIAamp viral RNA minikit (Qiagen). ARTIC v3 primer-based multiplex PCR amplicons were generated and used as the sequencing libraries (4, 5). Libraries were barcoded using the Oxford Nanopore Technologies native barcoding system (EXP-NBD104 and EXP-NBD114), pooled, and sequenced with a FLO-MIN106D flow cell (R9.4.1) for 6 h. Raw reads were base called and demultiplexed with MinKNOW v21.02.1. Processed reads were assembled with the ARTIC guppyplex code script with Medaka v1.4, using the ARTIC EPI2ME v3.3.0 SARS-CoV-2 pipeline (FastQC plus ARTIC plus NextClade) (https://artic.network/ncov-2019/ncov2019-bioinformatics-sop.html). To summarize, 3,723,478 reads were generated (range, 95,728 to 645,221 reads per sample; average length, 503 bp; read depth, 1,200× to 4,000×). A nearly complete genome was obtained for each sample, and the genomic information is provided in Table 1. SARS-CoV-2 lineages were assigned according to the proposed nomenclature of pangolin lineage assignment software v2.0.7 (https://github.com/cov-lineages/pangolin), followed by visual inspection using CLC Genomics Workbench v21.0 (Qiagen). In comparison with the reference genome (Wuhan Hu-1 [GenBank accession number NC_045512.2]), the spike protein-based signature amino acid variations assigned 22 of the sequences as sublineage BA.5 and 1 as BA.2.75. Genome sequencing has been playing a critical role in COVID-19 responses, because new variants are constantly evolving. Therefore, rapid sequencing and data sharing can contribute to the implementation of quick and informed public health decisions to mitigate COVID-19 consequences.

**TABLE 1:** Data for Bangladesh SARS-CoV-2 Omicron strains

Sample	Date of sample collection (day/mo/yr)	Patient age (yr)	Sex^*a*^	Symptoms^*b*^	Vaccination type	Booster dose completed	Pangolin lineage	GC content (%)	SRA accession no.	GenBank accession no.
TND-04-1538	29/5/2022	53	F	+	Oxford/AstraZeneca	No	BA.5.1	40.03	SRR20936503	OP164783
TND-04-1566	4/6/2022	26	M	+	Moderna	No	BA.5.1	40.36	SRR20936502	OP164784
TND-01-0668	8/6/2022	44	F	+	Oxford/AstraZeneca	Yes (Pfizer-BioNTech)	BA.5.3.1	39.77	SRR20936520	OP164786
IR-2986	13/6/2022	39	M	−	Oxford/AstraZeneca	No	BA.5.3.1	40.19	SRR20936521	OP164777
IR-2989	13/6/2022	77	M	+	Oxford/AstraZeneca	Yes (Moderna)	BA.5.3.1	41.02	SRR20936510	OP164778
TND-04-1606	13/6/2022	33	F	+	Not vaccinated	No	BA.5.2	39.95	SRR20936501	OP164785
IR-2991	14/6/2022	35	M	+	Oxford/AstraZeneca	Yes (Moderna)	BA.5.3.1	39.79	SRR20936507	OP164779
TND-07-1525	14/6/2022	55	M	+	Oxford/AstraZeneca	Yes (Moderna)	BA.5.3.1	39.59	SRR20936522	OP164776
TND-06-0758	14/6/2022	25	M	+	Pfizer-BioNTech	Yes (Moderna)	BA.5.3.1	40	SRR20936519	OP164787
TND-04-1610	14/6/2022	27	F	+	Oxford/AstraZeneca	No	BA.5.3.1	40.37	SRR20936518	OP164792
TND-04-1611	14/6/2022	69	M	+	Oxford/AstraZeneca	No	BA.5.3.1	40.56	SRR20936517	OP164793
TND-04-1612	14/6/2022	42	M	+	Sinopharm	No	BA.5.3.1	40.95	SRR20936516	OP164794
IR-2993	15/6/2022	35	F	+	Oxford/AstraZeneca	No	BA.5.3.1	40.17	SRR20936506	OP164780
TND-05-0773	15/6/2022	19	M	+	Pfizer-BioNTech	No	BA.5.2	40.89	SRR20936515	OP164795
TND-04-1623	16/6/2022	22	M	+	Sinopharm	No	BA.5	40.15	SRR20936514	OP164788
TND-04-1625	16/6/2022	37	M	+	Oxford/AstraZeneca	No	BA.5.3.1	40.92	SRR20936513	OP164796
TND-04-1628	16/6/2022	26	M	+	Sinopharm	No	BA.5.3.1	40.83	SRR20936512	OP164797
IR-2995	17/6/2022	29	F	+	Oxford/AstraZeneca	Yes (Moderna)	BA.5.2	39.95	SRR20936505	OP164781
IR-2998	18/6/2022	73	M	+	Oxford/AstraZeneca	Yes (Pfizer-BioNTech)	BA.5.3.1	40.04	SRR20936504	OP164782
TND-06-0768	18/6/2022	60	F	+	Sinopharm	No	BA.5.3.1	40.03	SRR20936511	OP164789
TND-06-0770	18/6/2022	66	F	+	Sinopharm	No	BA.5	40.76	SRR20936509	OP164790
TND-09-0721	18/6/2022	36	M	+	Not vaccinated	No	BA.5.3.1	39.87	SRR20936508	OP164791
TND-04-2046	8/7/2022	41	M	+	Sinopharm	No	BA.2.75	40.38	SRR21484218	OP390081

a F, female; M, male.

b +, present (fever, cough, and/or mild weakness); −, absent.

### Data availability.

The data from this study can be found under GISAID accession numbers EPI_ISL_13439470, EPI_ISL_13439471, EPI_ISL_13439472, EPI_ISL_13439473, EPI_ISL_13439474, EPI_ISL_13439475, EPI_ISL_13439476, EPI_ISL_13439477, EPI_ISL_13439478, EPI_ISL_13439479, EPI_ISL_13439480, EPI_ISL_13439481, EPI_ISL_13574266, EPI_ISL_13574267, EPI_ISL_13574268, EPI_ISL_13574269, EPI_ISL_13439482, EPI_ISL_13574270, EPI_ISL_13574271, EPI_ISL_13439484, EPI_ISL_13439485, EPI_ISL_13439486, and EPI_ISL_14859273. The GenBank accession numbers are listed in Table 1.
